# Attenuation of ischemia-reperfusion injury in swine resuscitated for hemorrhagic shock by low-dose inhaled nitrite or carbon monoxide

**DOI:** 10.1186/cc13309

**Published:** 2014-03-17

**Authors:** H Haugaa, H Gomez, D Maberry, A Holder, O Ogundele, A Botero, D Escobar, L Gordon, S Shiva, C Dezfulian, B Kenney, TI Tønnessen, B Zuckerbraun, MR Pinsky

**Affiliations:** 1 Oslo University Hospital, Oslo, Norway; 2 University of Pittsburgh Medical Center, Pittsburgh, PA, USA

## Introduction

Fluid resuscitation of hemorrhagic shock is frequently associated with reperfusion injury and secondary organ damage.

Studies suggest that low doses of both nitrite and carbon monoxide may protect tissues and organs from this reperfusion injury by limiting mitochondrial free radical production. We explored the effects of very small doses of nitrite and carbon monoxide on tissue injury in a porcine model of hemorrhagic shock.

## Methods

Fluid resuscitation of hemorrhagic shock is frequently associated with reperfusion injury and secondary organ damage. Studies suggest that low doses of both nitrite and carbon monoxide may protect tissues and organs from this reperfusion injury by limiting mitochondrial free radical production. We explored the effects of very small doses of nitrite and carbon monoxide on tissue injury in a porcine model of hemorrhagic shock.

## Results

Although no increase in blood nitrite concentrations was observed after inhalation, nitrite was associated with significant decreases in blood, muscle, and peritoneal fluid lactate concentrations (*P *< 0.05), whereas both nitrite and carbon monoxide were associated with significant decreases in glycerol in peritoneal fluid (*P *< 0.05). Following resuscitation, the muscle mitochondrial respiratory control ratio was preserved in the nitrite and carbon monoxide groups and reduced in the control group. Adjuvant drugs had no effects on any gross hemodynamic parameters.

## Conclusion

We conclude that at low doses, nebulized sodium nitrite and inhaled carbon monoxide are associated with tissue protection during resuscitation from severe hemorrhagic shock. Acknowledgement This work was supported in part by DoD grant W81XWH-11-2-0049, NHLBI HL07820, and 2013121 from The SouthEastern Norwegian Health Authorities.

